# Notes on the Application of Statistics, to Questions in Medical Science, Particularly as to the External Causes of Diseases

**Published:** 1855-12

**Authors:** W. P. Alison

**Affiliations:** M.D., Edin., D.C.L. Oxon., Emeritus Professor of Practice of Medicine, Edinburgh


### RECORD OF MEDICAL SCIENCE.

*Notes on the Application of Statistics, to Questions in Medical Science, particularly as to the External Causes of Diseases.* By W. P. ALISON, M.D., Edin., D.C.L. Oxon., Emeritus Professor of Practice of Medicine, Edinburgh.*

It has so frequently and so plausibly been urged, against all the inquiries and studies which are termed Statistics, that the form of such reasoning may be applied to the support of almost any proposition, that

* Read to the Statistical Section of the British Association at Glasgow, Sept. 1855 (by Sir Archibald Alison.)

it becomes an object of very considerable importance,—in the view of any one who is truly convinced of the importance and frequent practical application of such inquiries,—to point out the circumstances of any question, or departments of any science, in which this kind of information is truly requisite, and the conditions under which it may be trusted. This is especially true of the science of Medicine, because there is one great department of that science,—that which we term Etiology, or the doctrine of the external causes of diseases, in which our knowledge is acquired almost entirely in this way. It is in very few cases only, that our knowledge of the essential or intimate nature, either of diseases or of the powers in Nature which excite them, enables us to form any anticipation of the effects of those powers; and it is simply by *empirical* observation,—facts observed and recorded, and the frequency of their recurrence noted, although not explained, *i. e.*, it is by the mere *force of numbers*, or by *statistics*, whether stated exactly in that form or not, —that our information on that subject, and practical rules for the prevention or treatment of diseases, founded on that information, are acquired.

I have elsewhere stated,* what seem to me to be sufficient reasons, for the application of Statistics to inquiries of this kind being more frequently and obviously required, than in any of those which are made, either into the nature of diseased actions (or of vital actions in general,) or the power and mode of action of remedies. The questions which we propose to ourselves in Etiology are truly *simpler* than either in Pathology or Therapeutics,—they involve little or no exercise of judgment, simply the observing and recording of facts, according to directions which are made known, and the sufficiency of which can easily, at any subsequent time, be estimated;—the sources of fallacy connected with them are *less numerous*; and, more especially, we can usually remark this, as to the observations by which we can fix on those antecedents of a disease, to which we ascribe the power of producing it,—that the *positive observation*, of the alleged effect following the application of the cause, is supported by a large body of *negative observations*, often not stated in words, but truly essential to the validity of the inference; and, therefore, this class of observations is often more truly and efficiently statistical,—the number of individual cases really contributing to the result obtained, is very much greater, and the evidence afforded more decisive, than it can easily be made in the other departments of medical inquiries; sometimes, therefore, greater than those who have accustomed themselves only to physiological or pathological inquiries, or to watching the effects of remedies, can easily perceive.

The importance of this observation will not be denied, when we remember that this is the department of Medical science which is truly of the greatest practical importance. The knowledge of the external causes of diseases, is that which leads most directly to their prevention; and to the preservation of those lives especially, from which the greatest amount

* British and Foreign Review, etc. January and April 1854.

of labor of all kinds may be obtained, and which are, therefore, generally regarded as most valuable to a state.

In the course of the present century, improvements have been made in Medicine, which will bear comparison in their practically beneficial tendency, with those which have made this age and this quarter of the globe so illustrious, as regards the applications of any other Sciences to practical purposes. These have been almost exclusively in this department of medicine, and may be truly said to rest, as yet, almost exclusively on Statistics; anticipating, probably by several ages, any information within the power of the human race, as to the intimate nature of the phenomena which are thus recorded. It is simply by the force of numbers, attesting the simple fact of a disease, easily recognised, showing itself in many persons within narrow limits of time and space, and there only, that we have learnt how the poison of Small-pox may be diffused; and that it may be disarmed of its power over the human body, by being first taken into, and modified by its passage through, the body of the cow, and then applied, in almost infinitesimally small quantity, to the human body itself, and there exciting a certain specific inflammatory process, absolutely devoid of danger, and incapable of communication through the medium of the air, and, what is still more inconsistent with the knowledge we have of other changes in nature, that this modifying and preserving effect on the human body, produced in infancy, continues equally perfect, in a great majority of cases, after 60, 70, or 80 years, if life shall continue so long,—*i. e.*, after we are certain that the body on which it has been produced, has been repeatedly *worn down and built up again*, so that the poison introduced into the structure in advanced life, can no more be said to come in contact with living matter which has gone through the process of vaccination in infancy, than, according to the paradox of an ancient logician, a man can be said to have bathed twice in the same water, because he has bathed twice in the same river.

So also it has been simply empirical observation, and, therefore, by reference to statistics, that we have acquired within these few years, information touching the extension of another epidemic, less frequent, indeed, but attended with peculiar interest, and often with extreme fatality, the Puerperal fever, which enables us, with almost absolute certainty, to predict that its propagation, after the manner of an epidemic, may hereafter *always be prevented.* For, I believe, I may safely assert, that the statistical observations of Dr. Semmelweiss, in the great Lying-in-Hospital at Vienna, where upwards of 6000 births take place in a year,—being in exact accordance with what has been seen in this country, have unequivocally shown:

1. That this disease is, in fact, a case of the Diffuse inflammation or Erysipelas, attended with the same peculiarities in the extension of the inflammation, and in the nature of the effusion, and the same variation as to the nature of the accompanying fever in different epidemics.

2. That one immediate exciting cause of this form of inflammation, which may always be suspected when it prevails epidemically, is the *cadaveric* poison,—often evolved, during the decomposition of the human body, from whatever cause death may have taken place; but during one stage of that decomposition only, viz., that early stage during which the *post mortem* inspections for the investigation of the cause of death are most frequently made.

3. That when this form of inflammation assumes the form of epidemic puerperal fever, the mode of its transmission from one patient to another, is by accoucheurs or nurses, themselves in good health, but to whose persons or clothes minute portions either of the *effluvia from others* already affected, or of this *cadaveric poison*, have become attached; and

4. That when the obvious precautions are taken, to prevent any persons to whom, in either of these ways, the poison can have thus adhered, from acting as accoucheurs or nurses until effectually purified, no "epidemic extension" of puerperal fever is seen.

It is seldom, no doubt, that this last proposition can be submitted to a searching scrutiny, as in the year 1846, at the Vienna Hospital, under Dr. Semmelweiss, when the number of deaths after these precautions were adopted, was diminished by no less than 400 in that hospital alone, and in a single year; but when it is added, that each of the propositions above stated is quite in accordance with observations made and recorded (although the bond of connection among them had unfortunately not been duly observed) in this country, whenever puerperal fever has been epidemic, whether on a large or small scale,—so that it would be easy to collect statistical evidence of the same kind from every such epidemic, wherever any record of the facts has been left, to establish each of these propositions,—the force of this statistical evidence becomes such as to justify the sanguine anticipation above announced.

Our information as to the mode of extension of the malignant Cholera, is, unfortunately, as yet less certain or precise; yet I think we may say it is so far advanced, that we may entertain a confident *hope* of its being very soon such as to disarm any epidemic cholera, in this climate, at least, of all its terrors.

I allude to that subject here, chiefly because I think it affords an example worthy of notice, of what I have already stated, as to the frequent misapprehension of the strength of evidence, simply empirical, or founded on statistics, which may often be obtained in inquiries of this kind.

I mentioned formerly in a paper on this subject, which I had the honour of laying before this Association at Birmingham, a *single case* which I saw in the year 1832, and which I have always maintained to have been sufficient, for any one who duly attended to the statistical evidence it afforded, to establish the proposition, that the disease is capable of propagation in this climate, by the intercourse of the sick with the healthy, without pledging us to any opinion as to the mode of communication, or as to the existence of other modes. Now that it is generally admitted, that the assertion then made was correct, the question for discussion now being (as stated by the Editor of the *British and Foreign Medical Review*), "*not* whether cholera is contagious or not, but *how often* it spreads *by the agency of human bodies* (*i. e.*, by *contagion*), and how often without that agency," (Journal for January 1854, p. 298.)

I think myself justified in drawing attention to the grounds on which it was made; and which, I still think, amply sufficient to establish that mode of communication, without, of course, excluding others.

The reason of this strong expression of opinion was, that the case furnished a remarkable example of the evidence which, according to what was formerly stated, a single *positive* fact may afford,—quite of the nature of the *instantia crucis,* when supported by a large body of *negative* observations. The anxious expectation of the disease in the town, in 1832, the careful division of the town into districts, and appointing of stations and medical men to each, and the number, zeal, and intelligence of these observers, were known to be such as to justify our saying, that this was the very *first* case of the disease which ever originated in Edinburgh or Leith, in a person who had not left the town; and that for a time it was *an isolated* case, no other appearing for ten days, in a population of above 140,000 persons, many of whom were in circumstances, in all other respects at least, equally favorable to the appearance of Cholera, as this woman was;—as the subsequent appearance of the disease in many of them, during nearly a year that the disease afterwards existed in Edinburgh, proved. If the poison producing this "*nova pestis,*" had no contagious property, this woman was not more exposed to it than any other of this large body of people; but if it had that property—in whatever other way it might be communicated,—she was undeniably and peculiarly exposed, as she was engaged in nursing her son, in a close confined room, who was ill of the symptoms of cholera, during the whole of the day preceding that in which she sickened and died; and he had passed the next preceding night in a house in Musselburgh, where patients in the malignant disease then were, and had been for weeks previously. Here, then, was a fair *instantia crucis,* to determine whether the transmission of the disease, from place to place, known to be at that time a frequent event, was or was not dependent on intercourse of healthy persons, with those previously sick of it; and the affection of this one person, who had that intercourse, contrasted with the non-appearance of the disease in the 140,000 was, as I maintain, *decisive* evidence.

Many cases, equally decisive, have since occurred, of which I have been informed, on the *first* appearance of a disease *previously unknown* in a town or district, carefully observed;—*e. g.*, at Arbroath in 1853, where there was clear proof that the two first persons, of 15,000 inhabitants of that town, who took Cholera, "had just returned from Dundee, where they had visited persons ill of cholera;" their affection, therefore proves nothing as to the efficacy of intercourse with the sick, in exciting the disease, rather than merely visiting a particular locality;— but the next *six* who took it in Arbroath,—the *first six* inhabitants of that town who took cholera without leaving the town, had repeated and close intercourse with those already affected before they took it; and the inference as to the contagious property is drawn, not from their taking the disease, nor even from their taking it at that time, and in rapid succession, but from their being *the only inhabitants* of Arbroath who took it, during the first week of its existence in that town;—*i. e.*, the positive evidence of the *six* who were known to be exposed to that cause, is supported by the *negative* evidence of the *fifteen thousand* who could not be shown to be, and the immense majority of whom certainly were not, so exposed.

I have great hopes that we shall soon have statistical evidence to establish a proposition which no other evidence known to us could justify our admitting, but which, if established, would not only reconcile most of the conflicting statements on this subject, but serve as a guide to almost complete security from any epidemics of this disease in future; viz., that the poison of cholera, *like other known animal poisons,* is developed during the decomposition of the animal matter, the appearance of which is most characteristic of the disease,—that which constitutes the *"rice water stools,"*—but only in a *particular stage* of that decomposition, not immediately after its formation in, or discharge from, the body; and again, not after the decomposition has gone to a certain length. All this can be proved only by Statistics, but is quite susceptible of proof in that way. The last proposition above stated—that no poisonons property is attached to any part of a body dead of cholera, after a certain stage of decomposition has been passed, seems nearly ascertained from the number of instances in which dissecting-rooms have been supplied with bodies of persons dead of cholera, for many weeks together, without any of the students attending them being affected. That the peculiar matter of the cholera evacuations, during its decomposition, perhaps especially in dry air, has a peculiarly poisonous quality, was, I believe, first suspected by Liebig, and partly by the analogy of the poison already mentioned, causing erysipelatous inflammation and puerperal fever,—and also of the *sausage poison,* repeatedly observed on a large scale in Germany, which is developed during a certain stage of the decomposition of the animal matter of those sausages, and disappears when their putrefaction has so far advanced. That the cholera can be communicated to animals by inoculation with this matter, of the peculiar rice water stools, has been sufficiently proved by the experiments of Dr. Lindsay in this country; and if a few more experiments shall give results similar to those of M. Thiersch, at Munich, I think we may assert that the proposition above stated, is *statistically* proved. "Dr. Thiersch collected the intestinal contents, or the evacuations, of cholera patients, and let them decompose under the influence of air and heat. From day to day he dipped into this matter pieces of filtering paper, which he dried, for subsequent experiments on white mice. Two of these animals at a time were exposed to infection for four days, by having a square inch of the filtering paper, thus prepared, moistened with water and mixed with their food. Each mouse took thus 1-2000th gr. daily. The results were as follows:—The preparations from the matter during the first day of decomposition were *innocuous.* To this succeeded a period of from six to nine days, during which decomposition went on, and preparations from the matter in this second period of decomposition, caused disease in 30 out of 34 animals, and death in 12 of the 34. The symptoms were peculiar and characteristic. The hair fell off, the ears dropped, there was languor, then discharge from the bowels, first of white, then watery matter, the urine lost its smell, then was suppressed, the appetite became *depraved,* so that the animals would fill their stomachs with wool; there was no apparent sickness, but such tonic muscular contractions that they seemed dead some time before death. On dissection, accumulation of blood in the vessels of the small intestines was invariably found, their contents *watery*, with *abundant epithelial flakes* (just similar to those found in persons dead of cholera); the cortical substance of the kidneys passing into fatty degeneration, the bladder empty, the blood and the contents of the intestines answering to the test of ready mixture with amygdalin, as in the cholera of man,—which will not appear in the healthy animal. To this a third period of decomposition of the matter under trial succeeded, in which these poisonous effects were very slight, or not observed at all."—(See *Medical Times* and *Gazette*, Nov. 25, 1854.)

We have already a statement by Dr. Budd, of statistical observations made on villages in England, where the entrance of Cholera appeared to be prevented by such expedients as these observations immediately suggest,—for receiving the rice water evacuations of cholera patients on linen or cotton, and burying or burning, or otherwise effectually destroying their substance, during that period, thus indicated, after they have been passed, and before they have entered on the morbific decomposition; and we have sufficient statistical evidence of the importance of another measure,—which was first adopted, I believe, at Edinburgh in 1832, and has since been recommended by the Board of Health in London, and adopted in different places, although not so generally or satisfactorily as could be wished,—founded on the merely empirical statements I have made as to the communication of the disease from the sick to the healthy, and its apparent adhesion to particular, often very limited, localities, viz.,—the establishment of *houses of refuge* for the reception of all inhabitants of houses or rooms which might become infected with cholera in any town; not themselves affected, nor required for the care of the first cases that might occur. Here such persons might be lodged in pure air, regularly fed, preserved from cold and from other (frequently concurrent) exciting causes of the disease, and treated with due attention to cleanliness, immediately on any symptoms of cholera showing themselves. The London Board of Health report, that they had information of 1691 persons taken into these houses of refuge, from rooms where there were patients in cholera, and of these, only 33 became affected with cholera, and only 10 died. In cases of which I was myself informed, in Edinburgh, at Glasgow, and at Oxford, during different epidemics, 1010 persons were admitted from sick rooms into such houses of refuge, of whom 40 took the disease, and 15 died; whereas the experience of Dr. Hamilton of Falkirk, of 251 cases of cholera appearing in 86 houses, where no such means of separation existed, gives only a fair idea of the extent to which successions of cases will often be observed, in confined air and dirty districts, possessing no such resource.

I am happy to say, that so far back as November last, having written to Dr. A. Smith, at the head of the medical department of the army, on the subject of the decomposition of the rice water stools, as the probable cause of the progagation of cholera, and of districts becoming *tainted* with the poison of that disease,—I was informed by him that he had directed the attention of the medical officers in the Crimea to the facts now stated, so that if the disease shall appear in a malignant form in that army, we may hope for at least accurate and truly statistical information as to the reality of that opinion.

Again, as to the Yellow Fever, so frequently becoming epidemic in the hot climates, although we cannot boast of having acquired information either as to the nature of its cause, the essential character of the morbid change it produces, or the power of any remedy over it,—yet by simply empirical observations, *i. e.*, by Statistics, we have information to the following effect, as stated in reports in Germany and France, on inquiries conducted by order of those governments, on a large scale, and considered by committees containing the names of Humboldt and Dupuytren, that it is a disease *endemic*, almost exclusively, "in districts nearly on the level of the sea, never appearing beyond 48° of north latitude, nor without a previous temperature of 72°,—only in certain circumstances propagated by contagion," but when epidemic, always *confined strictly to certain localities;* so that the practical rule of immediately evacuating, *i. e.*, removing *all* the inhabitants of places where it is declared to exist, and has formerly prevailed, is *incontestable*, and "of such proved utility, as will always justify its rigorous execution."

Without dwelling farther on the effects of Malaria, in this or any other climate, which we can only expect to be satisfactorily explained when pathology shall be considerably more advanced than at present, I may merely add, that such collections of facts have been made, and are now frequently repeated, as to the places and circumstances in which it arises from the earth, and the laws according to which it extends and multiplies,—considered merely empirically or statistically,—as we may confidently expect to be successful in disarming this cause of disease likewise of its terrors, long before the nature of the change produced by its action on the living body, or the rationale of any line of treatment of those who may be affected by it, shall become known.

The cure of Epidemic Scurvy, resulting in different cases, as is now satisfactorily established, from different deficiencies in the Diet habitually taken,—the efficacy, therefore, of different kinds of diet in counteracting this form of disease,—still more remarkably, the power of small quantities of vegetable Acids in producing the same effect, and the extraordinary rapidity with which such changes of diet, and these acids, will produce their effect,—may also be stated as examples, on a large scale, and of the most satisfactory kind, of what is generally called the power of Art over a most loathsome and virulent disease,—but in reality must be regarded, now and probably long after our time, as results obtained by simply empirical observations of the course of Nature, statistically arranged, fortunately facilitated by so many of the subjects being organised bodies of men;—and which have distinguished the present age to a degree, which those who are not familiar with the medical writings of the last century will hardly conceive.

We have good reason to hope, that inquiries now on foot as to the external causes of Scrofulous, or of what is now usually called Tubercular disease, including pulmonary Consumption,—an inquiry which we must perceive to be more complex, and in which the operation of various causes must be recognised,—will be effectual in pointing out the means of counteracting that tendency in a very large proportion of cases of persons liable to it,—simply on the principle of empirical observation, enlarged and arranged in the form of Statistics, long before we shall have information as to the essential nature of the vital process, or mode of operation of the causes in question.

In illustration of this, I need only mention two facts, recently ascertained on so large a scale, that we have no doubt of their truth and importance, and which, even at present, may be said to be guides to successful practice in many cases only recently thought hopeless, although all that was previously known on the subject was certainly rather adverse than favorable to the supposition that they would ever be established. These are,—1. The good effect of the Cod-liver oil—if not of other animal Oils, on many cases of tubercular disease, in their early stage—provided only that it can be retained on the stomach to the extent of an ounce and a half or two ounces daily; and, 2. The almost complete exemption of the inhabitants of the Faro Islands from tubercular disease, notwithstanding that their climate, as regards cold and damp, is exactly that which, in this country, has been thought most favorable to it.—*Edinburg Med. and Surg. Journ.*

We regret that our limits will not permit us to copy the whole of Prof. Alison's interesting article. His concluding remarks are upon the operation of the New Poor Law Act.—Ed.

